# Simultaneous Extraction and Determination of Compounds With Different Polarities From Platycladi Cacumen by AQ C_18_-Based Vortex-Homogenized Matrix Solid-Phase Dispersion With Ionic Liquid

**DOI:** 10.3389/fphar.2018.01532

**Published:** 2019-01-09

**Authors:** Mingya Ding, Jin Li, Shuhan Zou, Ge Tang, Xiumei Gao, Yan-xu Chang

**Affiliations:** ^1^Tianjin State Key Laboratory of Modern Chinese Medicine, Tianjin University of Traditional Chinese Medicine, Tianjin, China; ^2^Tianjin Key Laboratory of Phytochemistry and Pharmaceutical Analysis, Tianjin University of Traditional Chinese Medicine, Tianjin, China; ^3^Department of Nephrology, The First Teaching Hospital, Tianjin University of Traditional Chinese Medicine, Tianjin, China

**Keywords:** AQ C_18_, ionic liquid, Platycladi Cacumen, UHPLC, vortex-homogenized matrix solid-phase dispersion

## Abstract

This study presented a rapid, simple and environmentally friendly method of employing AQ C_18_-based vortex-homogenized matrix solid-phase dispersion with ionic liquid (AQ C_18_-IL-VHMSPD) for the extraction of compounds with different polarities from Platycladi Cacumen (PC) samples by ultra high-performance liquid chromatography with PDA detection. AQ C_18_ (aqua C_18_) and ionic liquid ([Bmim]BF_4_) were used as the adsorbent and green elution reagent in vortex-homogenized MSPD procedure. The AQ C_18_-IL-VHMSPD conditions were optimized by studying several experimental parameters including the type of ionic liquid, the type of adsorbent, ratio of sample to adsorbent, the concentration and volume of ionic liquid, grinding time and vortex time. The recoveries of the target compounds were in the range of 96.9–104% with relative standard deviation values no more than 2.8%. The limits of detection and limits of quantitation were in the range of 0.2–1.2 and 1.0–5.4 ng mL^-1^, respectively. Compared with the traditional ultrasonic-assisted extraction, the developed AQ C_18_-IL-VHMSPD method required less sample, reagent and time. It was concluded that the AQ C_18_-IL-VHMSPD method was a powerful method for the extraction and quantification of the high polarity and low polarity compounds in traditional Chinese medicines samples.

## Introduction

**FIGURE 1:**
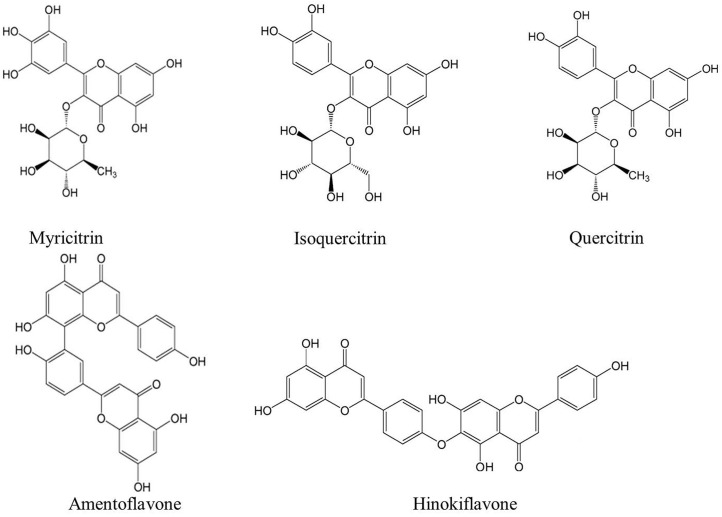
The chemical structures of five compounds assayed in Platycladi Cacumen sample.

Platycladi Cacumen (PC), namely Cebaiye, derived from the dry twigs and leaves of *Platycladus orientalis* (L.) Franco, is one of the most commonly used traditional Chinese medicines (TCMs). It has been applied in many TCM formulations for thousands of years. It was recorded that the PC could cool the blood to stanch bleeding, dispel pathogenic wind, remove dampness and resolve phlegm (Chinese Pharmacopoeia Commission, 2015). Recently, it was confirmed that PC has antimicrobial, anti-tumor, anti-inflammatory, anti-oxidant activities and neuroprotective effect (Hassanzadeh et al., 2001; Emami et al., 2011; Zaugg et al., 2011; Fan et al., 2012; Zhang et al., 2013). Flavonoids are regarded as the material basis for the efficacy. The main flavonoid glycosides consists of myricitrin, isoquercitrin, and quercitrin. In addition, there is a large amount of biflavonoids in PC. Among these biflavonoids, hinokiflavone, and amentoflavone are the representative ones (Lu et al., 2006). These five flavonoids with different polarities have been reported to exert a therapeutic effect on diseases in previous literatures (Ma and Wu, 2013). Thus, the complete extraction and precise analysis of the five compounds (Figure 1) with different polarities are particularly crucial for quality control and pharmacological investigations of herbal PC.

The conventional methods for the extraction of PC included the ultrasonic-assisted extraction (UAE) with HPLC-UV or UPLC-DAD (Zhuang et al., 2017, 2018), the microwave-based extraction with ultraviolet–visible detection, and heat reflux extraction with UPLC-DAD (Chen et al., 2007; Shan et al., 2018). However, these methods normally require a great deal of organic reagent, which arouse the environment pollution. Besides, the longer extraction time was another shortage. Matrix solid-phase dispersion (MSPD) is a comprehensive sample preparation method in which sample homogenization, disruption, extraction, fractionation and purification were simultaneously performed in one step and a shorter extraction time and less organic solvent were required (Barker et al., 1989; García-López et al., 2008). At present, many MSPD methods have been successfully applied for various samples (Liu et al., 2008; Vela-Soria et al., 2014). Recently, a vortex-homogenized MSPD (VHMSPD) technique not only retained the superiority of the traditional MSPD method but also overcame the shortage of the loss of analytes in complex procedures (Du et al., 2018a,b). However, no information about VHMSPD was available for the analysis of Platycladi Cacumen in the literature.

The adsorbent used in the MSPD procedure acts a pivotal part in improving the extraction efficiency of target analytes (Cao et al., 2016). Most applications of MSPD have utilized the silica gel matrix material and absorptive matrix materials. PSA, NH_2_, CN, COOH, C_8_, C_18_ (end capped), C_18_-N (no end capped) and AQ C_18_ were silica gel matrix material. Florisil was absorptive matrix materials. Compared with C_18_, more silanol groups linking to the surface of C_18_-N, which could provide the additional polar interactions. A certain proportion of polar functional groups were added to the surface of non-polar materials of AQ C_18_, which not only has the higher adsorption capacity on low polarity target compounds, but also greatly increases the adsorption capacity of high polarity target compounds. Thus, AQ C_18_ could be used to extract high polarity and low polarity target compounds from traditional Chinese medicines. To our knowledge, no reference on the use of AQ C_18_ as an adsorbent for the extraction of TCMs by VHMSPD has been reported.

Ionic liquids (ILs) are characterized by diverse combinations of organic or inorganic anions and organic cations. Recently, they have been widely used as green elution reagent for microextraction due to the advantages of high thermal stability, negligible vapor pressure and good solubility for inorganic and organic compounds (Han et al., 2012). It was reported that ILs could interact with analytes by various mechanisms such as π-π, electrostatic force, hydrogen bonding, ion-dipole, inclusion complex etc. (Qiu et al., 2012). Thus, the green ILs were applied for extracting analytes using VHMSPD method in the TCMs.

In this study, an AQ C_18_-based vortex-homogenized matrix solid phase dispersion with ionic liquids (AQ C18-IL-VHMSPD) technique with UHPLC-PDA was first established for simultaneous determination of compounds with different polarities from Platycladi Cacumen including three high polarity flavonoid glycosides (myricitrin, isoquercitrin and quercitrin) and two low polarity bioflavonoids (hinokiflavone and amentoflavone). The combination of AQ C_18_ and ionic liquid was applied to extract natural compounds in the vortex-homogenized matrix solid phase dispersion procedure. Some parameters such as type of adsorbent, sample/adsorbent ratio, and type and concentration of eluent were optimized to obtain a good extraction efficiency in detail. Furthermore, the five compounds were extracted by using the conventional ultrasonic-assisted extraction to evaluate the feasibility of the developed AQ C_18_-IL-VHMSPD method.

## Materials and Methods

### Chemicals and Reagents

Reference standards of myricitrin, isoquercitrin, quercitrin, amentoflavone and hinokiflavone were purchased from Chengdu Desite Bio-Technology Co., Ltd., (Chengdu, China). PSA (40–60 μm, 60 A), NH_2_ (40–60 μm, 60 A), CN (50 μm, 60 A), COOH (50 μm, 60 A), Florisil (60–100 μm, 80 A), C_8_ (50 μm, 60 A), C_18_ (50 μm, 60 A), C_18_-N (50 μm, 60 A) and AQ C_18_ (50 μm, 60 A) were supplied from Agela Technologies. 1-butyl-3-methylimidazolium tetrafluoroborate ([Bmim]BF_4_), 1-hexyl-3-methylimidazolium tetrafluoroborate ([Hmim]BF_4_), 1-octyl-3-methylimidazolium tetrafluoroborate ([Omim]BF_4_), 1-butyl-3-methylimidazolium hexafluorophosphate ([Bmim] PF_6_) and 1-octyl-3-methylimidazolium hexafluorophosphate ([Omim]PF_6_) were purchased from Shanghai Chengjie Chemical Co., Ltd., HPLC-grade acetonitrile and methanol were purchased from Dikma Technologies Inc., United States. HPLC-grade formic acid was purchased from Tedia Company, Inc. (Tedia, Fairfield, OH, United States). Deionized water was purified from a Milli-Q academic ultra-pure water system (Millipore, Milford, MA, United States). Other chemical reagents were of analytical grade. All the solutions were filtered through a 0.22 μm filter membrane before UPLC analysis.

### Plant Material

A total of 8 batches of Platycladi Cacumen and its processed products were collected from different regions of China and authenticated by Dr. Yan-xu Chang (Tianjin University of Traditional Chinese Medicine). All samples were pulverized using a pulverizer (Zhongcheng Pharmaceutical Machinery) after being dried at 60°C for 24 h, then passed through a 100-mesh sieve.

### Preparation of Standard Solutions

The standard stock solutions of myricitrin, isoquercitrin, quercitrin, hinokiflavone and amentoflavone were separately prepared in methanol. Myricitrin and quercitrin were 2 mg mL^-1^. Isoquercitrin, hinokiflavone and amentoflavone were 1 mg mL^-1^. The appropriate amount solution of all standards was diluted with methanol to obtain eight different appropriate concentrations for calibration curves. The concentrations of myricitrin, isoquercitrin, quercitrin, hinokiflavone and amentoflavone were in the range of 0.4–100, 0.08–20, 0.8–200, 0.2–50, and 0.2–50 μg mL^-1^, respectively. The related standard solutions were stored at 4°C.

### Ultra High-Performance Liquid Chromatography With PDA Detection (UHPLC-PDA) Analysis

**FIGURE 2:**
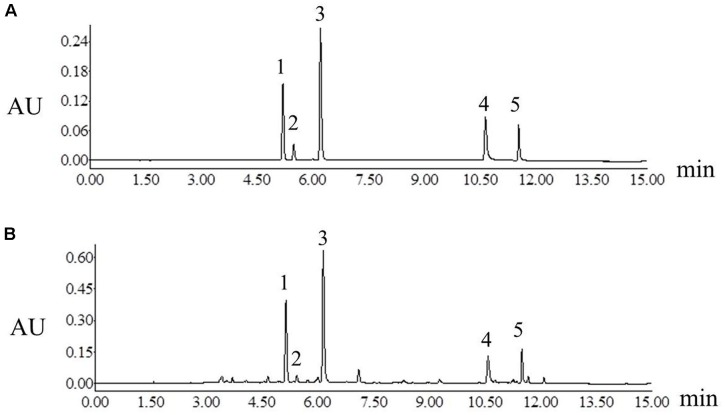
Ultra high-performance liquid chromatograms of mixture of standard compounds **(A)** Platycladi Cacumen samples **(B)**. Peak: 1, myricitrin; 2, isoquercitrin; 3, quercitrin; 4, amentoflavone; 5, hinokiflavone.

The chromatographic analysis was performed on a Waters ACQUITY UPLC System (Waters Co., Milford, MA, United States) that consisted of a photodiode array (PDA). The workstation controlled by Empower 2 software was employed to collect and analyze data. The separation was performed on an ACQUITY UPLC BEH C_18_ column (2.1 mm × 100 mm, 1.7 μm, Waters) at the flow rate of 0.3 mL min^-1^. The mobile phase consisted of water with 0.1% formic acid (eluent A) and acetonitrile (eluent B) using a gradient elution: 0–2 min, 5–37% B; 2–9 min, 37–67% B; 9–10 min, 67–85% B; 10–13 min, 85–95% B; 13–15 min, 95–5% B, then post run 6 min. The column temperature was maintained at 30°C and the injection volume was 1 μL. The detection wavelength was set at 340 nm. Under the above chromatographic conditions, the chromatographic peaks of analytes included samples and standard solutions were separated excellently (Figure 2).

### AQ C_18_-Based Vortex-Homogenized Matrix Solid-Phase Dispersion With Ionic Liquid Procedure

**FIGURE 3:**
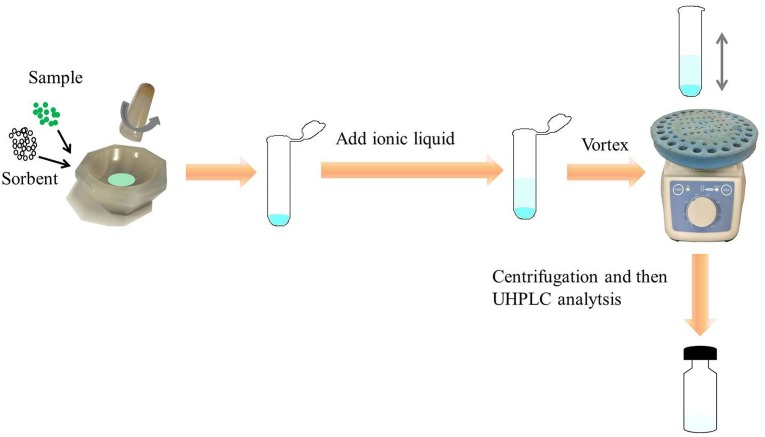
Schematic diagram of AQ C_18_-based vortex homogenized MSPD with ionic liquid method.

An aliquot of 25 mg of the previously crushed sample and 50 mg adsorbents (AQ C_18_) were put into an agate mortar gradually. The mixture was grinded with a pestle for 3 min. Once completely dispersed, the mixture was transferred into a 4 mL polypropylene tube. 1.5 mL elution reagent ([Bmim]BF_4_) was added and then thoroughly shaken by vortex for 45 s. Subsequently, the tubes were placed into a centrifuge at 14000 rpm for 10 min. The supernatant liquor was collected and 1 uL was injected into the UHPLC for analysis. The schematic diagram of AQ C_18_-IL-VHMSPD method was exhibited in Figure 3.

### Ultrasonic Extraction

According to the Chinese Pharmacopoeia 2015, the dried PC samples (0.500 g) were precisely weighed and introduced into a 100 mL Erlenmeyer flask, then mixed with 20 mL methanol. Finally the mixture was extracted ultrasonically (40 kHz, 96% power) for 30 min and the weight loss of the solution was complemented with methanol. All the extract solution was filtrated through a 0.22 μm filter membrane and 1 μL filter liquor was injected into the UPLC-PDA for further analysis.

### Optimization of AQ C_18_-IL-VHMSPD Parameters

To obtain a good extraction efficiency of the target compounds, several experimental parameters including the type of adsorbent, ratio of sample to adsorbent, type and concentration of the eluting solvent, and grinding time were investigated. Each test was repeated in triplicate.

Several adsorbents were investigated including PSA, NH_2_, CN, COOH, Florisil, C_8_, C_18_, C_18_-N and AQ C_18_. An aliquot of 25 mg PC samples and 50 mg adsorbents were transferred into an agate mortar, then grinded for 3 min. The eluent was 1.5 mL 90 mM ILs and 3 min was chosen as vortex time. Different types of ILs such as [Bmim]BF_4_, [Hmim]BF_4_, [Omim]BF_4_, [Bmim]PF_6_ and [Omim]PF_6_ were optimized. Then, four levels of concentration of [Bmim]BF_4_ (50, 70, 90, and 110 mM) and volumes of IL (0.5–2 mL) were considered to be optimized. The ratio of sample to adsorbent (1:0, 1:1, 1:2, and 1:3) was considered to investigate while the other conditions remained unchanged. In addition, the vortex time (15, 30, 45, and 60 s) and grinding time (0, 1, 2, 3, and 4 min) were also tested.

## Results and Discussion

### Optimization of the AQ C_18_-IL-VHMSPD Method

#### Type of Adsorbent

**FIGURE 4:**
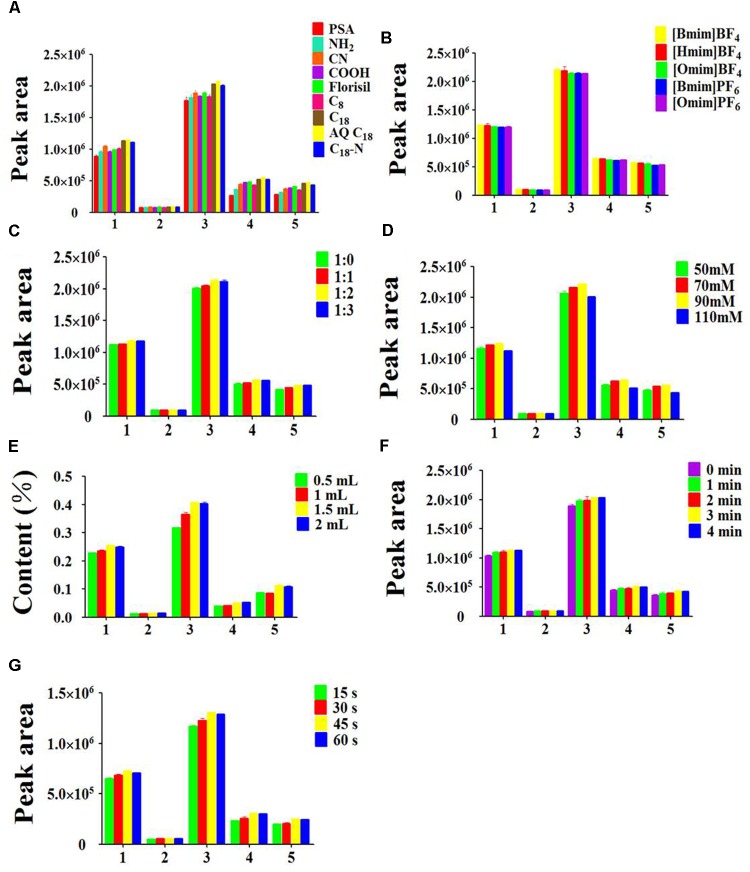
Effects of parameters on extraction efficiency of five peak: (1) myricitrin, (2) isoquercitrin, (3) quercitrin, (4) hinokiflavone, (5) amentoflavone, **(A)** type of the adsorbent, **(B)** type of the ionic liquid, **(C)** ratio of sample to adsorbent, **(D)** ionic liquid concentration, **(E)** ionic liquid volume, **(F)** grinding time, **(G)** vortex time. The errors bars represent RSD (*n* = 3).

In the development of the MSPD procedure, it is crucial to employ a suitable dispersing adsorbent. The adsorbent is not only used as a disruption and dispersion agent that destroys the structure of samples for the extraction of the target compounds, but also as a purificant that removes the interfering substance of matrix. In the present study, nine types of adsorbent were evaluated. As Figure 4A shows, the analytes has the strongly retention when PSA, NH_2_, CN, COOH, Florisil and C_8_ were used as adsorbent. The reason was that the analytes bonded with the adsorbent too tightly to be eluted effectively. C_18_-N and C_18_ produced the equivalent extraction efficiency, but not dramatically better than AQ C_18_. One probable explanation was that the abundant Si-O-Si and Si-OH groups formed hydrogen bonds between adsorbent and analytes. Additionally, the interaction force may be supplied by the silica, including hydrogen bonding and electrostatic interaction. Therefore, AQ C_18_ was chosen as the optimal adsorbent for the subsequent extraction procedure.

#### Type of Ionic Liquid

An appropriate type of elution solvent is a significant parameter to obtain good extraction efficiency for target analytes. The elution solvent should have a similar polarity as target analytes, which is easy to disrupt the interaction between the target analytes and the adsorbent (García-Mayor et al., 2012). [Bmim]BF_4_, [Hmim]BF_4_, [Omim]BF_4,_ [Bmim]PF_6_ and [Omim]PF_6_ are five common ionic liquids. To select the most appropriate one, these five ILs were evaluated based on the extraction efficiency of the target analytes in the MSPD procedure. As shown in Figure 4B, the elution efficiency of ILs was affected by the alkyl chain length of cation and the types of anion. It was quite obvious that the peak areas of the five target compounds were significantly increased from [Omim]BF_4_ to [Bmim]BF_4_. The mechanism of this phenomenon is that the hydrogen bonding interaction was weakening between the target analytes and imidazolium rings when alkyl chain length was increased with the same anion BF_4_^-^. Although [Bmim]BF_4_ and [Bmim]PF_6_ had the same cation, the highest peak areas of all analytes were observed with the BF_4_^-^ anion. The possible reason is that the combination of anion BF_4_^-^ and analytes generated the stronger electrostatic interaction. Overall, [Bmim]BF_4_ was chosen as the optimum elution solvent for the next experiment.

#### Ratio of Sample to Adsorbent

The mass ratio of adsorbent to sample is a significant parameter affecting the extraction yield of the target analytes from the samples (Rodrigues et al., 2010; Xu et al., 2016). An appropriate ratio of sample to adsorbent not only guarantees that the sample is completely homogenized and dispersed in the adsorbent, but also decreases the loss of sample in the blending procedure. It can be seen in Figure 4C that the peak areas of the five target compounds were distinctly increased when the sample/adsorbent ratio increased from 1:0 to 1:2. The possible reason was that the larger the amount of adsorbent, the stronger the molecular interaction produced, such as hydrogen bonding and electrostatic force between analytes and adsorbents. However, further increment of the sample/adsorbent ratio led to a slightly decrease of the extraction efficiency. The reason may be that the excessive adsorbent generated such a strong interaction between the five compounds and AQ C_18_ that eluent could not completely eluted. Thus, sample/sorbent ratio of 1:2 was selected.

#### Concentration and Volume of Ionic Liquid

The concentration and volume of [Bmim]BF_4_ are also crucial parameters affecting the extraction yield of the target analytes in the elution process. The results (Figure 4D) showed that the peak areas of target compounds were gradually increased from 50 to 90 mM. A possible reason was that the π-π, electrostatic and hydrogen-bond interactions between the [Bmim]BF_4_ and target analytes were stronger than the interaction of the analytes and adsorbent, while the extraction efficiency was slightly decreased following the increment of the concentration of [Bmim]BF_4_. This phenomenon could be ascribed to the intensive viscosity, which gave rise to poor capacity to transfer the target analytes from adsorbent into eluent. Thus, 90 mM [Bmim]BF_4_ seemed to be the optimum concentration for further experiments.

To attain the highest extraction efficiency with the minimum volumes of ILs, 0.5 mL to 2 mL volume of 90 mM [Bmim]BF_4_ were investigated. Figure 4E demonstrates that the contents of the five compounds were increased from 0.5 to 1.5 mL. The reason could be that the stronger interaction was generated between analytes and eluant along with the increase of the volume of eluant. Nevertheless, the peak areas of the five compounds remained unchanged when the volume kept increasing. *T*-test was introduced to evaluate the differences between two groups by Microsoft excel (version 2010). The results showed that there was no significant difference between 1.5 and 2 mL of 90 mM [Bmim]BF_4_. Consequently, 1.5 mL of [Bmim]BF_4_ was used in the following MSPD extraction procedure.

#### Grinding Time

The grinding time is a vital parameter of great concern in the VH-MSPD method. In order to evaluate the effect of different grinding times, five time intervals at 0, 1, 2, 3, and 4 min were tested. As the results show in Figure 4F, the peak areas of the 5 target analytes were increased with increasing grinding time from 1 to 3 min. It was likely that the longer grinding time caused a stronger interaction force between AQ C_18_ and analytes, facilitating the transfer of the target analytes from samples into the adsorbent. However, the extraction efficiency employing 3 min-grinding was approximately equal to that of 4 min-grinding. Thus, the grinding time for 3 min was chosen for further experiments.

#### Vortex Time

Previous studies have proven that vortex time is an important factor influencing the extraction efficiency. As shown in Figure 4G, the peak areas of the five target compounds were significantly increased as the vortex time increased from 15 to 30 s. However, increasing the vortex time to 60 s resulted in a slightly decline of the peak areas. Thus, 45 s was chosen as the optimal vortex time. Eventually, the optimized AQ C_18_-IL-VHMSPD conditions were determined to be 25 mg PC sample, 50 mg AQ C_18_, grinding time of 3 min, 1.5 mL 90 mM [Bmim]BF4 as the elution solvent and 45 s vortex time.

### Method Validation

#### Selectivity and Linearity

The calibration curves (*n* = 8) of 5 analytes were obtained by performing the peak areas as Y-axis, versus the concentration in μg mL^-1^ as X-axis, which ranged from 0.08 to 200 μg mL^-1^. The correlation coefficients (R^2^) of each analyte were higher than 0.9997 (Table 1).

**Table 1:** Linearity, limits of detection (LOD), limit of quantification (LOQ), and repeatability of the proposed method (*n* = 6).

Compounds	Regressive equation	Linear range (μg/mL)	*R*^2^	LOD (μg/mL)	LOQ (μg/mL)	Repeatability RSD (%)
Myricitrin	*y* = 18697× + 4126.3	0.4–100	1	0.0003	0.001	1.9
Isoquercitrin	*y* = 20257× + 593.13	0.08–20	0.9999	0.0002	0.0054	0.9
Quercitrin	*y* = 18819× + 9536.7	0.8–200	0.9999	0.0002	0.001	1.3
Amentoflavone	*y* = 40153× – 1750.5	0.2–50	0.9997	0.0012	0.004	3.7
Hinokiflavone	*y* = 59804× + 2041.5	0.2–50	0.9999	0.0008	0.002	1.9


#### Limits of Detection and Quantification

Limits of detection (LOD) and limit of quantification (LOQ) were employed to assess the sensitivity of the developed method. They were estimated as the concentrations of the analytes when the signal-to-noise (S/N) ratio reached 3 and 10 individually. The LODs of the five compounds ranged from 0.0002 to 0.0012 μg mL^-1^, while the LOQs ranged from 0.001 to 0.0054 μg mL^-1^ (Table 1).

#### Reproducibility

The repeatability was evaluated by six parallel AQ C_18_-IL-VHMSPD extracts of a PC sample. As summarized in Table 1, the values of relative standard deviations (RSDs) were all less than 3.7%. It was confirmed from the results that the developed method had good reproducibility during experiment.

#### Precision, Stability and Recovery

Instrumental precision was expressed as intra-day and inter-day precision by determining the relative standard deviations (RSDs) at three levels of concentrations in six replicates of each compounds. Intra-day precision and inter-day precision were tested in a single day and within continuous 3 days, respectively. The validation results are presented in Table 2, the accuracies were within the range of 95.2–104.2% (RSD ≤ 3.3%) and 96.2–114.6% (RSD ≤ 3.1%) for intra-day precision and inter-day precision, respectively.

**Table 2:** The results of precision and stability.

Compounds	Concentration (μg/mL)	Intra-day		Inter-day		Stability	
		RSD (%)	Accuracy (%)	RSD (%)	Accuracy (%)	RSD (%)	Remain (%)
Myricitrin	1	1.4	96.9	1.7	96.2	1.4	98.2
	5	0.5	99.9	1.5	99.4	1.2	98.9
	25	1.4	101	2.1	101	0.9	101
Isoquercitrin	0.2	1.3	97.8	2.0	96.4	1.2	95.8
	1	0.9	98.5	0.9	98.2	1.4	97.6
	5	1.5	103	2.1	103	1.0	103
Quercitrin	2	1.3	97.4	1.7	97.4	1.3	95.4
	10	0.6	95.5	1.3	98.4	1.1	97.0
	50	1.5	95.2	2.2	96.4	0.9	96.4
Amentoflavone	0.4	0.8	113	2.7	115	2.6	115
	2	0.9	104	0.6	104	1.6	103
	10	3.3	104	3.1	104	1.8	104
Hinokiflavone	0.4	0.4	97.3	0.2	97.5	1.5	97.6
	2	0.4	100	0.5	101	0.8	100
	10	0.7	97.4	1.0	97.4	0.4	97.4


The stability was investigated by analyzing the accuracies of three levels of concentrations of five compounds at room temperature condition over 24 h. The accuracies of five compounds were in a range of 95.8–114.8%, while with the RSDs were both no more than 2.6%, showing that the analytes were stable from 0 to 24 h at room temperature.

To verify the accuracy of the proposed method, recovery tests were performed by analyzing the spiked sample in triplicate. Unspiked samples and spiked samples were simultaneously extracted using the optimum AQ C_18_-IL-VHMSPD procedures. The results are listed in Table 3. The mean recoveries of 5 compounds were all in the range of 96.9–103.6% and the RSDs were all less than 2.8%, which demonstrated that the proposed AQ C_18_-IL-VHMSPD method was reliable and effective.

**Table 3:** The results of recovery test (*n* = 6).

Compounds	Original (μg)	Spiked (μg)	Detected (μg)	Average recovery (%)	RSD (%)
Myricitrin	60.85	25	85.5	98.4	2.5
Isoquercitrin	4.34	2.5	6.8	96.9	2.8
Quercitrin	107.84	50	159.6	104	1.6
Amentoflavone	11.38	5	16.5	102	0.6
Hinokiflavone	6.30	1.25	7.5	98.4	1.9


### Application

All of the above analysis results demonstrated that the proposed method had applicable value. Thus, the developed AQ C_18_-IL-VHMSPD method was employed to analyze the target compounds with different polarities using the last-optimized conditions in six batches of crude Platycladi Cacumen and two batches of carbonized Platycladi Cacumen obtained from various producing areas. The contents of myricitrin, isoquercitrin, quercitrin, amentoflavone and hinokiflavone in crude PC were in the range of 1.64–2.10, 0.11–0.16, 3.41–4.13, 0.40–0.48, and 0.20–0.26 mg g^-1^, individually (Table 4). Furthermore, the contents of myricitrin, isoquercitrin, quercitrin, amentoflavone and hinokiflavone in carbonized PC were in the range of 0.06–0.17, 0.00–0.01, 0.03–0.25, 0.02–0.06, and 0.02–0.04 mg g^-1^, respectively. The results clearly revealed that the contents of the five target compounds were remarkably decreased after processing.

**Table 4:** Contents of the five flavonoids of Platycladi Cacumen samples from 8 batches (*n* = 6).

No.	Production region	Content (mg/g)				
		Myricitrin	Isoquercitrin	Quercitrin	Amentoflavone	Hinokiflavone
1^a^	Shandong	2.05 ± 0.01	0.16 ± 0.00	3.76 ± 0.03	0.44 ± 0.01	0.25 ± 0.01
1^∗^	Shandong	2.02 ± 0.00	0.16 ± 0.00	3.73 ± 0.01	0.46 ± 0.01	0.24 ± 0.01
2^a^	Anhui	2.03 ± 0.04	0.16 ± 0.00	3.83 ± 0.03	0.46 ± 0.01	0.25 ± 0.00
2^∗^	Anhui	1.99 ± 0.02	0.16 ± 0.00	3.82 ± 0.01	0.46 ± 0.00	0.26 ± 0.01
3^a^	Unknown	2.10 ± 0.04	0.16 ± 0.00	4.13 ± 0.04	0.46 ± 0.00	0.24 ± 0.00
4^a^	Unknown	1.64 ± 0.03	0.11 ± 0.00	3.41 ± 0.05	0.48 ± 0.01	0.26 ± 0.01
5^a^	Unknown	1.89 ± 0.02	0.15 ± 0.00	3.83 ± 0.01	0.40 ± 0.01	0.20 ± 0.00
6^a^	Unknown	2.01 ± 0.05	0.16 ± 0.00	3.76 ± 0.10	0.40 ± 0.01	0.20 ± 0.01
7^b^	Anhui	0.17 ± 0.01	0.01 ± 0.00	0.25 ± 0.03	0.06 ± 0.01	0.04 ± 0.00
8^b^	Unknown	0.06 ± 0.00	0.00 ± 0.00	0.03 ± 0.00	0.02 ± 0.00	0.02 ± 0.00


*^a^Crude Platycladi Cacumen samples; ^b^processed Platycladi Cacumen samples; ^∗^the certain Platycladi Cacumen sample was extracted by the authoritative extract method (Pharmacopoeia of China 2015).*

In order to compare the proposed AQ C_18_-IL-VHMSPD method with conventional ultrasonic-assisted extraction from Pharmacopoeia of China 2015, the contents of the five target compounds from the same batches of PC were determined by these two methods. It was found that there was no significantly difference between the contents of the five components by two methods. The results indicated that the developed AQ C_18_-IL-VHMSPD method had the almost same effectiveness as the method of Pharmacopoeia of China 2015 for extracting PC.

### Comparison With Other Methods

To evaluate the performance of the proposed AQ C_18_-IL-VHMSPD method, several methods including ultrasonic assisted extraction and reflux extraction were introduced to compare the sample amount, extraction solvent, solvent volume, extraction time and detection time. As summarized in Table 5, it is obviously observed that the developed AQ C_18_-IL-VHMSPD method has the lower extraction time and detection time in contrast with other methods. Except for the method 2 in Table 5 (deep eutectic solvents based ultrasonic assisted extraction) and the AQ C_18_-IL-VHMSPD method, other methods all employed a large volume of organic solvent. Moreover, the proposed method required less extraction time and detection time than deep eutectic solvents based ultrasonic assisted extraction method. Overall, these results indicated that the AQ C_18_-IL-VHMSPD method was a rapid, simple and environmentally friendly method for the extraction of the high polarity and low polarity compounds in PC samples.

**Table 5:** Comparison of the AQ C_18_-IL-VHMSPD method with other methods in the determination of compounds in Platycladi Cacumen sample.

No.	Extracted compounds	Sample amounts (mg)	Type of solvent	Solvent volume (mL)	Extraction method	Extraction time (min)	Detection method	Detection time (min)	Reference
1	Myricitrin, isoquercitrin, quercitrin, myricetin, afzelin, quercetin, kaempferol, amentoflavone, hinokiflavone	500	75% ethanol	10	UAE^a^	60	UPLC-DAD	24	Zhuang et al., 2018
2	Myricitrin; quercitrin; amentoflavone; hinokiflavone	25	Deep eutectic solvents	1	UAE^a^	30	HPLC-UV	28	Zhuang et al., 2017
3	Myricitrin, isoquercitrin, quercitrin, afzelin, cupressuflavone, amentoflavone hinokiflavone	1000	Ethanol–water (75:25)	50	Reflux	28	UPLC-DAD	30	Shan et al., 2018
4	Myricitrin, isoquercitrin, quercitrin, amentoflavone hinokiflavone	25	Ionic liquid	1.5	AQ C_18_-IL-VHMSPD	0.75	UPLC-PDA	15	This work


## Conclusion

An environmentally friendly sample pretreatment method, AQ C_18_-based vortex-homogenized matrix solid-phase dispersion with ionic liquid was successfully developed to extract and quantify the target analytes with different polarity from Platycladi Cacumen by UHPLC-PDA. AQ C_18_ was employed as the adsorbent to improve the adsorption capacity of compounds of different polarity. The use of green ionic liquids reduced the environment pollution. Compared with other extraction methods (UAE and reflux extraction), the present method is rapid, time-saving and efficient. This proposed method could be used for determination of compounds with different polarity from other traditional Chinese medicines.

## Author Contributions

Y-xC, GT, XG, and JL designed the experiments. MD and SZ performed the experiments. MD wrote the manuscript.

## Conflict of Interest Statement

The authors declare that the research was conducted in the absence of any commercial or financial relationships that could be construed as a potential conflict of interest.
